# Evaluation of Dynamic Foraminal Stenosis with Positional MRI in Patients with C6 Radiculopathy-Mimicking Pain: A Prospective Radiologic Cohort Study

**DOI:** 10.1155/2022/1385387

**Published:** 2022-06-09

**Authors:** Ozcan Kaya, Kerim Sariyilmaz, Yildiray Tutpinar, Mehmet Fevzi Cakmak, Mehmet Semih Cakir, Okan Ozkunt

**Affiliations:** ^1^Istanbul Kanuni Sultan Suleyman Training and Research Hospital Orthopedics and Traumatology, Kucukcekmece, Istanbul, Turkey; ^2^Acibadem Mehmet Ali Aydinlar University School of Medicine Atakent Hospital, Kucukcekmece, Istanbul, Turkey; ^3^Bahcesehir LIV Hospital, Department of Radiology Esenyurt, Istanbul, Turkey; ^4^Kirsehir Ahi Evran University, Orthopedics and Traumatology, Istanbul, Turkey; ^5^Acibadem Maslak Hospital Department of Radiology Sariyer, Istanbul, Turkey; ^6^Medicana Health Group Istanbul Bahçelievler Hospital Orthopedics and Traumatology Bahçelievler, Istanbul, Turkey

## Abstract

**Objective:**

Patients with a C6 radiculopathy-mimicking complaint are always in the gray zone if the diagnosis is not clear. The aim of the study is to make the diagnosis clear if the neck and shoulder pain is caused by a dynamic stenosis of the neural foramen at the C5-C6 level.

**Methods:**

Patients with a C6 radiculopathy-mimicking complaint were included in the study. Patients had a cervical spine magnetic resonance imaging (MRI) at the normal limits, or a minimal protrusion at the C5-C6 level underwent a dynamic MRI procedure. We measured the foraminal area and spinal cord diameter (SCD) at the C5-C6 level by using the PACS system ROI irregular are determination integral embedded to PACS. Inter- and intraobserver reliability of measurements was evaluated. Results were analyzed statistically, and a *p* value< 0.05 was accepted as statistically meaningful.

**Results:**

A total of 23 patients between January 2019 and June 2019 were included in the study. There were 10 men and 13 women, and the mean age was 41.3 (range 33-53). Foraminal area decrease at C5-C6 in extension and increase in flexion when compared with the neutral position was statistically significant (*p* < 0.001). Foraminal area changes between the complaint side and the opposite side was not statistically different (*p* > 0.05). Interobserver and intraobserver reliability of measurements were classified as in almost perfect agreement.

**Conclusions:**

Our present work presented dynamic and positional foraminal changes in MRI with radiculopathy-mimicking patients. Soever, we did not find a difference between the clinical complaint side and the opposite side in radiculopathy-mimicking patients. Cervical radiculopathy pain should not be attributed only to foraminal sizes. PACS embedded irregular area measurement integral allows the easy measure of a big number of patients without additional set-up and digital work requirements.

## 1. Introduction

During daily outpatient services, we assess too many patients with radiculopathy complaints caused by cervical discopathy. Commonly, the most severe level of stenosis and posterior bulging was found at the C5-C6 level. Although MRI is the standard imaging method for assessment of cervical cord/root compression, sometimes, despite exaggerated symptoms and physical examination of radiculopathy, we cannot put forth the evidence of disease with conventional magnetic resonance imaging (MRI), and in such cases, the standard imaging was insufficient to explore the exact pathology [1]. That is why several authors investigated dynamic MRI for cervical degenerative cases, obtained with the neck in different positions [2–7]. Previously, researchers studied this issue in different cohorts, and Zhang et al. reported the first study in 2011 that evaluated cervical spinal cord measurements by flexion-extension positional MRI in patients with previously known cervical spinal myelopathy [2]. It has been also shown that sagittal cervical motion affects foraminal dimensions, spinal cord length, and spinal cord volume at the cervical spine region in healthy subjects and cadavers [6–9].

Cervical nerve root impingement at the foramen is believed to be the cause of radiculopathy. Nerve root impingement due to foraminal stenosis also may result from a sequel of degenerative arthritis and disc collapse resulting from disc degeneration [9]. Some authors suggests that cervical radiculopathic pain also could be caused by ischemic changes within or around the neural tissue. Additionally, degenerated disc with osteophyte formation may cause venous obstruction and perineural fibrosis in the foramen which are thought to be related with the pathogenesis of radicular pain. The neural foraminal size was previously studied with different methods including CT, cadaveric measurements, and volume scanning techniques [9, 10]. According to our knowledge, our present study will be the first to explore the foraminal size by using positional MRI.

Patients who have typical radiculopathy symptoms and physical evaluation findings without MRI evidence established the present work's starting point. We designed this prospective cross-sectional study to evaluate with regular MRI these patients with unproven radiculopathy. We aimed to figure out unproven radiculopathy with positional MRI on these patients.

## 2. Materials and Methods

We hypothesized that dynamic cervical foraminal stenosis caused neck and shoulder pain which cannot be screened with routine MRI. Our study evolved from this idea and was designed as a prospective cohort study. Patients who were evaluated with dynamic cervical MRI for unknown C6 root compression and radiculopathy in 2019 were included in the study prospectively. For evaluating foraminal diameter change, the control group is defined as regular MRI of patients. Exclusion criteria were previous cervical spine surgery, neoplasia, severe cervical spinal stenosis, contraindications for MRI acquisition, and those who declined study participation. The study protocol was approved by the Hospital Ethics Committee and International Review Board before initiation, and informed consent was obtained from all patients.

Patient sample pool was created according the inclusion criteria: (1) typical complaints of C6 radiculopathy; (2) clearance from shoulder, neck, and chest region disorders with physical examination and shoulder MRIs; and (3) regular MRI without obvious vertebra foraminal stenosis (positional MRI revealed decrease of foraminal area compared with regular MRI foraminal diameter size) and degenerative disc disease (degenerative disc degeneration which did not make significant compression to nerve roots in the neural foramen).

### 2.1. Dynamic Magnetic Resonance Imaging Protocol

All patients underwent an MRI examination of the cervical spine using the same device (1.5 Tesla, Ingenia Philips, Koninklijke, Netherlands) under supervision of one of the study authors. During the first step, the examination was conducted with the patient in the routine supine position with the neck in the neutral position to obtain conventional T1- and T2-weighted sagittal and axial sequences. In the second step, MRI acquisition was performed with a collar to provide maximal flexion and extension positions. Patients were instructed to immediately interrupt the examination if they experienced any discomfort or neurological complaint. The MRI acquisitions in the flexion and extension positions only included T2-weighted sequences in the sagittal plane to help minimize the examination time. All patients were evaluated for potential neurologic deficits or discomfort before and after MRI procedures.

### 2.2. Imaging Analysis and Morphometric Parameters

The imaging analysis was performed using the Synapse software with a standardized 200% zoom, by two independent observers (YT and MSC). After 2 weeks, one observer (MSC) performed the measurements in the same manner.

**Figure 1:**
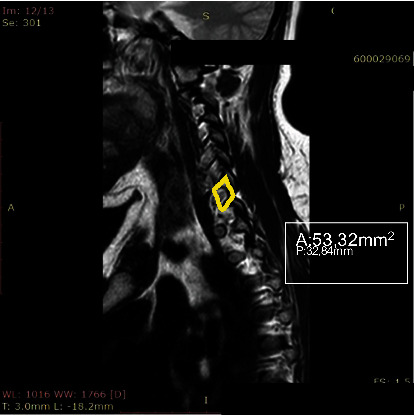
Measurement of foraminal area was performed with automated integral calculations embedded to PACS.

**Figure 2:**
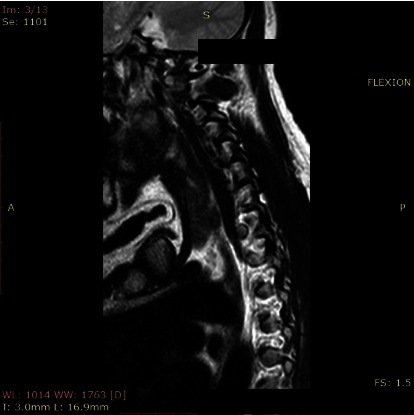
Flexion view of C5-C6 foramen at the largest diameter.

**Figure 3:**
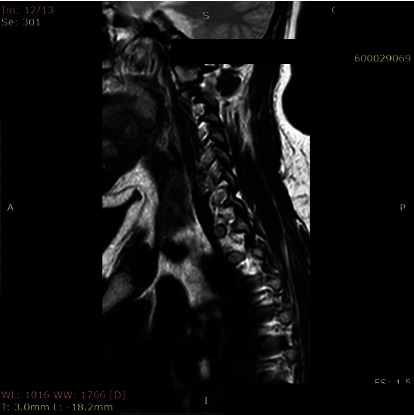
Neutral view of C5-C6 foramen at the largest diameter.

**Figure 4:**
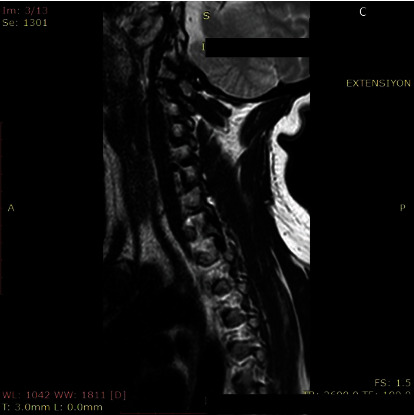
Extension view of C5-C6 foramen at the largest diameter.

The morphometric parameters considered for the analysis were divided into two methods. First, we measured the foraminal area (FA) and then the spinal canal diameter (SCD) at the disc level. The foraminal area was measured as the adjacent superior and inferior vertebral pedicles; the posteroinferior margin of the superior vertebral body, the posterior intervertebral disc, and the posterosuperior margin of the vertebral body were used as anterior boundaries, with the ligamentum flavum and superior and inferior articular facets serving as posterior boundaries. Measurement of FA was performed with automated integral calculations embedded to PACS (Figure 1). Foramens were visualized at the highest diameter when the axial scan passed through the middle of the spinous process. In rotator positions, MRI scans were directed perpendicular to foramens to receive the keyhole foraminal figure (Figures 2, 3, and 4).

The SCD was measured as the distance between the midpoint of the posterior portion of the intervertebral disc and the anterior margin of the ligamentum flavum posteriorly. In the C5-C6 level, all parameters were measured in millimeters at the midline images of the T2-weighted sequence in the sagittal plane in the neutral, flexion, and extension positions.

### 2.3. Statistical Analysis

Statistical analyses were performed using SPSS statistical software version 24.0 (SPSS Inc., Chicago, IL). Data analysis and confirmation of distribution were performed with the Kolmogorov-Smirnov test. Differences between the neutral position and flexion and between the neutral position and extension were analyzed using a paired *t* test.

The intra- and interobserver reliabilities of the MRI morphometric parameter measurements were quantified using the intraclass correlation coefficient (ICC), with a confidence interval of 95%. ICC values of 0.00 to 0.20 were considered as slight agreement, 0.21 to 0.40 as fair agreement, 0.41 to 0.60 as moderate agreement, 0.61 to 0.80 as substantial agreement, and 0.81 to 1.00 as almost perfect agreement [11].

## 3. Results

A total of 23 patients with a suspicion of C5-C6 foraminal stenosis presenting to outpatient service between January 2018 and June 2018 were included in the study. There were 10 men and 13 women, and the mean age was 41.3 (range 33-53). All patients completed MRI procedures, and none of patients had any neurological complaints.

**Figure 5:**
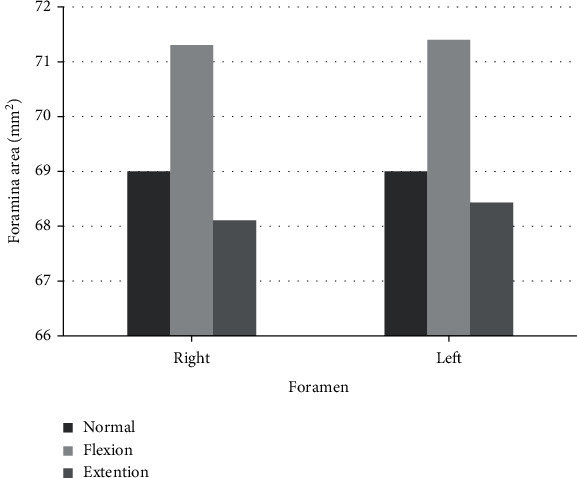
The mean foraminal area at different positions were outlined in the diagram.

The C5-C6 foraminal area and SCD were analyzed bilaterally at the neutral, flexion, and extension positions (Table 1). The mean foraminal area at different positions is outlined in Figure 5. Foraminal area decrease at C5-C6 in extension and increase in flexion when compared with the neutral position was statistically significant (*p* < 0.001). Foraminal area changes between the complaint side and the opposite side were not statistically different (*p* > 0.05). Interobserver and intraobserver reliabilities of measurement were classified as almost perfect agreement.

The spinal cord diameters at the foraminal level were statistically decreased with extension when compared with the neutral position (*p* = 0.035), but in flexion, the spinal cord diameter change is not meaningful statistically (*p* = 0.369). For the SCD measurement, interobserver and intraobserver reliance was classified as substantial agreement in all positions.

## 4. Discussion

Foraminal stenosis results in nerve root impingement at the foraminal level and causes radicular pain pattern and clinical findings [1]. Here, important points are rooming of the root at the foramen and size of the borders. During daily activities, some patients complain of typical radicular pain, but routine cervical MRI of patients do not put forth root irritation or impingement of roots despite clinic signs. The aim of present study was to evaluate foraminal stenosis if present under sagittal motion with positional MRI which was in the gray zone within these patients. Previously, in vivo and in vitro with small numbers of patients and cadaver samples, it has been shown that foraminal diameters increase with flexion and decrease with extension [9, 10]. In accordance with literature knowledge, we found that the foraminal area increased with flexion of the cervical spine and decreased with neck extension.

Foraminal area narrowing is caused by several factors. Degenerative arthritis and disc protrusion are common causes of foraminal stenosis and nerve root impingement. It is believed that decrease of disc height causes change in adjacent foraminal dimensions additional to the previously diminished foramen with a protruded disc [10]. Uncovertebral joint osteophytes or protruded disc may also cause root impingement despite unaffected foraminal dimensions. More recently, Le Vasseur and friends presented that degenerative changes in older ages are more related with foraminal dimension decrease in their in vivo study [12].

The initial mechanism of cervical radiculopathy-related pain is not clear; it is generally thought that in addition to mechanical compression of the nerve root, ischemic changes including nerve root and surrounding tissue may play an important role in generating cervical radiculopathy pain [13]. There is also evidence that diminished venous flow in the foramens may occur which results to perineural fibrosis and pain [14]. Farmer and Wisneski concluded that increasing neck extension led to significant pressure change in the nerve roots as a cause of radicular pain [15]. Besides these hypotheses, the dorsal ganglion size which is greatest at the cervical level also contributes to nerve compression [16]. Evaluating all these factors, nerve root compression results from interactions of the foraminal dimension, nerve position in the foramen, and nerve root size [9]. We did not find statistical difference between the symptomatic side and the opposite side, and this finding supports the importance of these aforementioned interactions.

Nerve root compression caused by foraminal stenosis can be evaluated by MRI, but at clinical settings, false negative and false positive rates are high [9]. The standard approach for the cervical MRI study is a supine-lying patient without weight bear. This position may not accurately represent the severity of foraminal stenosis, and many physicians think that foraminal dimensions are altered by changes in position of the cervical spine [3, 9, 10].

Our patient cohort had shoulder and neck pain. They had been evaluated for other systemic and local disorders which can cause radiculopathy-mimicking pain, and cervical MRI of patients were at the normal limits. MRI studies were accepted as false negative, and positional MRI studies were performed. In accordance with literature, we found the flexion position resulted in increase in the foraminal area and extension resulted in decrease of the foraminal areas [3, 9, 10]. In our cohort, we did not find difference between patients' painful side and opposite side for foraminal areas (*p* > 0.05). The spinal cord diameter with positional MRI was not different in extension and flexion compared with the neutral position. This proved the homogeneity of the patient group without obvious degenerative changes.

In the literature, different methods have been used to evaluate the foraminal size in the cervical spine. More recently, a 3D model-based matching technique from MRI images or biplane radiography systems [12, 17] was used. Previously, in vitro cadaveric studies were based on clipper or probe measurement techniques of foramens [10, 15]. Kitagawa et al. used CT images for evaluation of foraminal areas, but they pointed to inability of the CT scan to show soft tissues around the nerve root [9]. Here, in our study, we used the PACS system, and measurement of FA was performed with automated integral calculations which are used for irregular area calculations. Inter- and intraobserver reliability analyses were meaningful and in accordance with the literature. Here, CT evaluation is thought to be the best method for osseous foraminal walls, but soft tissue surrounding the nerve root should be taken into account because, at some points, soft tissue pressure changes and ischemic processes may be the cause of the radiculopathic pain [15]. It seems logical to explain radiculopathic pain when opposite foraminal diameter changes did not differ from each other at positional MRI.

Our study has several limitations when looking at many points. First of all, it is a diagnostic study, but we could not put forth the exact cause of radiculopathy-mimicking pain in our patients because the clinical side and the opposite side did not differ from each other in terms of the foraminal area. Only one level of foraminal evaluation can be criticised, but the patient cohort was designed for looking for a specific gray zone complaint. The number of patients would be accepted as too small to make a definite conclusion. Our patients underwent positional MRI in the supine position without load bearing, but our advantage was the rigid orthosis to protect the cervical position during MRI. As stated above, the three-dimensional structure of the foramen makes it hard to evaluate with available technologies, but the results of different methods show consistency with the literature.

## 5. Conclusion

Our present work presented dynamic and positional foraminal changes in MRI with radiculopathy-mimicking patients. Soever, we did not find a difference between the clinical complaint side and the opposite side in radiculopathy-mimicking patients. Anatomic measurement results of our study are compatible with the literature. More studies with larger cohorts would present dynamic changes especially in gray zone patients. The PACS embedded irregular area measurement algorithm gives an easy measure of a big number of patients without additional set-up and digital work requirements. Studies introducing the pathomechanism of radiculopathic pain and foraminal changes in these patients would improve understanding and develop treatment algorithms.

## Figures and Tables

**Table 1:** Summary of measurements in terms of foraminal area and spinal cord diameter at C5-C6 level.

Measurement	Right FA (mm^2^)	Left FA (mm^2^)	SCD (mm)	FA right vs. left
*Neutral*				
Mean	67.80	65.93	11.04	
Range	52.35-93.61	52.06-95.53	9.4-13.6	
Sd	±12.92	±12.31	±1.53	
*Flexion*				*p* > 0.05
Mean	74.1	71.67	11.30	
Range	55.32-98.98	53.6-101.4	9.81-14.54	
Sd	±14.19	±13.69	±1.57	
*p* value	<0.001	<0.001	0.369	
*Extension*				*p* > 0.05
Mean	62.69	62.48	10.65	
Range	50.23-87.41	50.11-89.8	8.67-13.1	
Sd	±11.49	±11.69	±1.58	
*p* value	<0.001	<0.001	0.035	

FA: foraminal area; SCD: spinal canal diameter; Sd: standard deviation.

## Data Availability

Due to the prospective nature of the study, data sharing is restricted by the local institutional review board.
